# Psychosocial and structural barriers to dual careers among Korean student-athletes: a multilayered ecological perspective

**DOI:** 10.3389/fpsyg.2025.1588430

**Published:** 2025-08-13

**Authors:** Seong Jun Ha

**Affiliations:** Department of Physical Education, Korea National University of Education, Cheongju, Republic of Korea

**Keywords:** student-athletes, dual career, sport-education interface, Korean sport system, ecological approach

## Abstract

**Introduction:**

This qualitative study investigates psychosocial and structural barriers to dual careers faced by Korean student-athletes. In South Korea, coexistence of the elite-centered sports culture and exam-driven education system poses significant challenges for student-athletes seeking to balance their academic and athletic goals.

**Methods:**

This study is grounded in Bronfenbrenner’s ecological systems theory and explores interactions across five ecological levels: microsystem (parents and coaches), mesosystem (school-sport relationships), exosystem (institutional structures), macrosystem (sociocultural norms), and chronosystem (policy timing). Data were collected through in-depth, semi-structured interviews with 20 participants, including student-athletes, coaches, parents, and teachers. Thematic analysis was conducted using a multilevel coding process.

**Results:**

Five core themes emerged: (1) narrow and fragmented career awareness influenced by close stakeholders, (2) passive compliance with sports-centered expectations in school and family systems, (3) structural constraints within athlete development and admissions systems, (4) sociocultural pressure stemming from South Korea’s competitive university entrance landscape, and (5) misalignment between dual-career policy intentions and field-level implementation timing.

**Discussion:**

These findings illustrate how systemic interactions across ecological levels restrict student-athletes’ abilities to pursue dual careers in South Korea. To address these barriers, this study recommends integrated policy strategies that promote institutional flexibility, inter-stakeholder collaboration, and cultural acceptance of diverse career pathways. This research provides practical insights for improving dual-career systems in centralized sports structures, drawing on global models such as the EU Guidelines on Dual Careers of Athletes.

## 1 Introduction

The concept of a dual career refers to the simultaneous pursuit of athletic performance and the development of academic or vocational competencies. It has recently gained attention as a key strategy in sport systems to promote athletes’ holistic development and improve their post-retirement quality of life (Aquilina, 2013; Capranica and Guidotti, 2016). A dual career offers several developmental benefits, such as enhanced athletic performance, psychological resilience, broader identity, social integration, and increased employability (Petitpas et al., 2009; Torregrosa et al., 2015; Sorkkila et al., 2017).

Governments and sports organizations worldwide have actively implemented policies to support dual careers. In Denmark, an ecological approach has been adopted to establish a structured, athlete-centered support environment (Henriksen et al., 2020), and the European Union has developed a formal policy foundation through its Guidelines on Dual Careers of Athletes (Capranica and Guidotti, 2016). Particularly, the COVID-19 pandemic accelerated the transformation of dual-career support systems, considering the widespread adoption of digital technologies that enable remote learning and hybrid training environments. Henriksen et al. (2024) interpreted unexpected crises such as the pandemic as transitions rather than disruptions, emphasizing the need for dual-career environments that can adapt flexibly to such changes. They argued that an effective design of transitional environments requires temporal sensitivity, structural connectivity, and cultural responsiveness through multilayered support systems. Furthermore, recent studies have stressed that dual-career environments should not be viewed as fixed institutional models; rather, they should be designed flexibly according to the cultural and organizational context of each country (Stambulova et al., 2015; Fusco et al., 2024; Henriksen et al., 2024).

Despite such efforts, dual career pathways still face structural barriers. These include academic overload, time constraints, rigid school systems, and insufficient institutional support (López de Subijana et al., 2015; Ryan, 2015). In Korea, the combination of a centralized elite sports system and a highly exam-oriented education culture restricts student-athletes’ academic engagement. Training and competition schedules take precedence over school participation, while career exploration and preparation receive little support.

Most research on dual careers has been conducted in Europe and North America. The European Union introduced comprehensive policies through its Dual Career Guidelines (Capranica and Guidotti, 2016), and the NCAA in the United States provides integrated support in partnership with schools (Gaston-Gayles, 2004). In these institutionalized systems, dual career experiences have shown positive impacts on athletes’ retirement transitions, employability, psychological well-being, and social adjustment (Murphy et al., 1996; Aquilina, 2013; Park et al., 2013). Finnish studies also found that dual career environments help student-athletes build a meaningful identity that integrates academic and athletic roles (Korhonen et al., 2020).

However, these findings largely reflect institutional contexts that differ from Korea. Elite sports still dominate Korean school systems, making direct application of these models inappropriate. A contextualized structural analysis is therefore necessary.

A recent study by De Maio et al. (2025) examined student-athletes’ perspectives on dual careers, identifying five key domains of disparity and implementation challenges: policy, financial support, academic flexibility, time management, and social support. The study proposed a multidimensional analytical framework that offers a valuable conceptual map for cross-national comparisons and policy development. Building on this approach, the present study explores the psychosocial and structural factors shaping Korean student-athletes’ dual career experiences.

To analyze these complex dynamics, this study adopts Bronfenbrenner’s ecological systems theory (Bronfenbrenner, 1979) as its analytical framework. The theory explains human development through interactions between individual characteristics and multiple environmental layers, including the microsystem (e.g., parents, coaches), mesosystem and exosystem (e.g., institutions, organizations), and chronosystem (e.g., career transitions). This framework is particularly useful for understanding the dual career experiences of student-athletes in a holistic, multilayered context.

The purpose of this study is to investigate the psychosocial and structural barriers faced by Korean student-athletes in their dual career pursuits. It analyzes the perspectives of student-athletes, coaches, parents, and teachers, and examines how their interactions and institutional settings affect the sustainability of dual careers. The study ultimately aims to identify strategies tailored to Korea’s sports and education systems and to offer practical recommendations for institutional and policy reform.

## 2 Theoretical framework

Bronfenbrenner’s (1979) Ecological Systems Theory offers an effective framework for analyzing student-athletes’ dual career experiences. The theory explains human development as shaped by interactions across multiple environmental systems. It consists of five levels: the microsystem, mesosystem, exosystem, macrosystem, and chronosystem (Bronfenbrenner, 2013). This ecological perspective aligns well with recent sport psychology research that highlights multidimensional and interactional views of athletes’ development and career transitions (Stambulova et al., 2021).

The microsystem involves environments where student-athletes engage directly, such as relationships with coaches, parents, teachers, and peers. These interactions provide essential emotional and psychological support in both academic and athletic contexts. The mesosystem includes interactions among microsystems—for example, communication between school staff and sports teams or between coaches and parents. These exchanges influence role conflict and daily experiences.

The exosystem comprises institutional and structural factors that affect student-athletes indirectly. These may include competition schedules set by federations, school policies, or parents’ work conditions. The macrosystem reflects broader cultural values, norms, and ideologies at the national or societal level, such as attitudes toward dual careers and elite sports. The chronosystem captures the temporal dimension of development, addressing changes over time such as school transitions, coaching shifts, or policy reforms.

This ecological model moves beyond single-factor explanations. It enables a comprehensive understanding of how diverse contextual factors shape and constrain dual career experiences. By applying this framework, the present study explores structural barriers and interactional dynamics within the dual career environment of Korean student-athletes.

## 3 Materials and methods

This study used a qualitative research design to examine the psychosocial and structural barriers faced by Korean student-athletes in pursuing dual careers and to identify relevant policy implications. The analysis focused on the experiences and perspectives of key stakeholders who play critical roles in the dual career ecosystem: student-athletes, coaches, teachers, and parents. Bronfenbrenner’s ecological systems theory served as the guiding theoretical framework for data analysis.

### 3.1 Participants

Participants were purposefully selected to reflect diverse perspectives within the dual career context. The sample included elite student-athletes (currently enrolled in middle or high school with at least 5 years of athletic experience), coaches (minimum of 10 years as athletes and 5 years as coaches), teachers managing school sports teams (with at least 5 years of experience), and parents actively involved in their child’s academic and career decisions.

A total of 20 participants were recruited through purposive sampling: six student-athletes, six coaches, four parents, and four teachers. To ensure variation in dual career experiences, participants were selected based on diversity in sport type (individual vs. team), geographic region (metropolitan vs. non-metropolitan), gender, and school level (middle vs. high school).

Sample size was determined by the principle of informational richness, a common standard in qualitative research. By involving stakeholders from different ecological levels—student-athletes, coaches, parents, and teachers—the study aimed to capture deep, context-rich insights. This approach aligns with the study’s goal of examining the multilayered structure and lived realities of dual career environments. General demographic characteristics of the participants are shown in Table 1.

**Table 1:** General characteristics of research participants.

School level	Gender	Experience (years)	Sports type
Middle School	Male	7	Football
	Female	5	Badminton
	Female	5	Table Tennis
High School	Male	8	Basketball
	Male	9	Baseball
	Female	10	Taekwondo
Middle School	Male	15	Football
	Male	17	Badminton
	Female	13	Table Tennis
High School	Male	8	Basketball
	Male	20	Baseball
	Female	19	Taekwondo
Middle School	Female	5	Badminton
	Female	5	Table Tennis
High School	Male	8	Baseball
	Female	10	Taekwondo
Middle School	Male	8	Football, Table Tennis
	Male	6	Volleyball, Badminton
High School	Male	11	Taekwondo, Table Tennis, Volleyball
	Female	5	Badminton

### 3.2 Data collection

Data were collected through in-depth interviews and document analysis. Individual interviews followed a semi-structured guide and lasted between 40 and 80 min. The interview protocol was adapted from the Dual Career Questionnaire developed by the Fédération Internationale du Sport Universitaire [FISU] and European Athlete as Student Network [EAS] (2016), and was modified to match the specific goals of this study.

All interviews were audio-recorded with participants’ consent and transcribed verbatim. To support contextual and interpretive analysis, researchers also documented emotional responses and non-verbal cues observed during the interviews. Sample interview questions are shown in Table 2.

**Table 2:** Examples of interview questions.

Examples of Interview Questions
What kinds of difficulties have you experienced in trying to balance your elite sports participation with your academic responsibilities? Are you familiar with any policies, programs, or initiatives that support the integration of elite sports and academic education? What forms of support does your school (or team) offer to elite athletes to facilitate the combination of sports and academic pursuits? Who supports your dual career at the level of your sport team or federation? Within your school, who is responsible for supporting your efforts to balance academics and elite sports? On a personal level, who provides support for your efforts to balance academics and elite sports?

### 3.3 Data analysis

This study adopted an interpretive approach to qualitative research to explore the psychosocial and structural barriers student-athletes face in dual career development within multilayered environments. The analysis followed Corbin and Strauss’s (2015) three-step coding procedure: open coding, axial coding, and selective coding. Bronfenbrenner’s ecological systems theory served as the guiding framework for organizing and interpreting the data (Tables 3–7).

**Table 3:** Coding summary: microsystem – fragmented career awareness.

Ecological system	Core theme	Sub-theme	Number of participants (*n*/20)	Representative quote (participant ID)
Microsystem	Fragmented Career Awareness	Perception that athletic performance is more advantageous than academic achievement for future careers	S: 4/6, C: 2/6, P: 1/4	“Even if I fully commit to sports, making it to the pros is uncertain—trying to do both feels like giving up.” (S1)
		Low motivation for academic engagement	S: 3/6, P: 1/4	“I feel like the time spent sitting in the classroom is meaningless.” (S5)
		Career Value Imposition by Coaches and Parents	C: 3/6, P: 2/4	“Student-athletes should focus on training rather than studying.” (C2)

S = Student-athlete, C = Coach, P = Parent, T = Teacher.

*n*/20 = Number of participants (out of 20) who mentioned the theme.

**Table 4:** Coding summary: mesosystem – compliance with avoidance of dual career.

Ecological system	Core theme	Sub-theme	Number of participants (*n*/20)	Representative quote (participant ID)
Mesosystem	Compliance with Avoidance of Dual Career	Passive and compliant communication among school, family, and coach	T: 3/4, C: 3/6, P: 2/4	“The requests are so strong that we excuse students from classes even if it’s not educationally appropriate.” (T3)
		Reduced academic engagement to secure training time	P: 3/4, C: 2/6	“Since sports are more important than studying, we inevitably reduce academic time.” (P1)

S = Student-athlete, C = Coach, P = Parent, T = Teacher.

*n*/20 = Number of participants (out of 20) who mentioned the theme.

**Table 5:** Coding summary: exosystem – structural issues in the student-athlete development system.

Ecological system	Core theme	Sub-theme	Number of participants (*n*/20)	Representative quote (participant ID)
Exosystem	Structural Problems in the Student-Athlete Development System	Performance-oriented structure in academic advancement and career decisions	C: 2/6, T: 2/4, P: 2/4, S: 2/6	“If there are no winning records, they cannot get into college. That’s why we increase training.” (T5)
		Institutional and competition schedule conflicts restricting academic participation	S: 3/6, T: 1/4	“When competitions are held during the semester, we inevitably have to skip classes.” (S1)
		Early specialization and lack of institutional support	T: 2/4, P: 1/4	“The system has been structured around sports rather than academics since an early age.” (T1)

S = Student-athlete, C = Coach, P = Parent, T = Teacher.

*n*/20 = Number of participants (out of 20) who mentioned the theme.

**Table 6:** Coding summary: macrosystem – intensified university entrance competition in Korean society.

Ecological system	Core theme	Sub-theme	Number of participants (*n*/20)	Representative quote (participant ID)
Macrosystem	Intensified University Entrance Competition in Korean Society	The need to enter a prestigious university for career opportunities after sports	S: 2/6, P: 3/4, T: 2/4	“Even if they cannot continue sports, they should at least graduate from a good university.” (P5)
		University entrance success perceived as a prerequisite for joining professional or corporate teams	P: 2/4, T: 1/4	“They need to go to a good school to be noticed by pro teams, or at least get a decent job later.” (T2)
		Social pressure and expectations for upward mobility through education	S: 1/6, P: 1/4, T: 1/4	“We also need to get into top universities like general students.” (S1)

S = Student-athlete, C = Coach, P = Parent, T = Teacher.

*n*/20 = Number of participants (out of 20) who mentioned the theme.

**Table 7:** Coding summary: chronosystem – temporal mismatch in dual career policy implementation.

Ecological system	Core theme	Sub-theme	Number of participants (*n*/20)	Representative quote (participant ID)
Chronosystem	Temporal Gap in Dual Career Policy Implementation	Confusion among the current athlete generation due to policy changes	P: 2/4, C: 2/6, T: 1/4	“They’ve only focused on sports until now, but suddenly they are told to attend all classes — they are the ones who suffer.” (P1)
		Limited university admission opportunities due to sudden changes in eligibility criteria	C: 2/6, T: 1/4	“If they do not meet the academic standards, they cannot compete or go to college — that does not make any sense.” (C4)
		Need for phased implementation to ensure effective policy integration	P: 1/4, T: 1/4	“It’s the right direction, but we need to proceed step by step.” (T4)

S = Student-athlete, C = Coach, P = Parent, T = Teacher.

*n*/20 = Number of participants (out of 20) who mentioned the theme.

All interviews were professionally recorded and transcribed verbatim. The researcher repeatedly reviewed the transcripts to identify underlying meanings in participants’ statements. In the open coding phase, meaningful units—words, phrases, or sentences—were segmented and coded. Codes were either recorded in participants’ own language (*in vivo* coding) or summarized with interpretive labels (descriptive coding). For example, the statement “I have to give up my studies to prepare for competitions” was coded as “perception of academic abandonment” and “compliance with training-first norms.” A total of 18 initial codes were generated, covering challenges such as time conflicts, coach-dominated structures, performance-driven parental attitudes, lack of career guidance, and institutional pressure.

During axial coding, the researcher analyzed logical relationships among the codes and grouped them into higher-order categories. This process identified causal links, conditions, and interaction patterns. For instance, “lack of communication between coaches and teachers” and “schedule conflicts between training and classes” were integrated into the broader theme of “structural disconnection between school and sports institutions.” Axial coding yielded nine core themes that represent structural factors influencing dual career engagement across ecological levels.

In selective coding, the core themes were reorganized according to the five levels of Bronfenbrenner’s ecological systems theory: microsystem, mesosystem, exosystem, macrosystem, and chronosystem. Aligning the empirical categories with this theoretical structure led to five overarching themes.

Theme development was guided not by frequency alone, but by recurring structural patterns and their meaning in the ecological context. Data saturation was reached after the 16th interview, when no new codes or themes emerged. The final four interviews confirmed the stability of the key themes. Peer debriefing and member checking verified the saturation judgment, enhancing the credibility of the findings.

The inclusion of 20 participants was theoretically justified. To represent the ecological model’s multiple levels, the study included student-athletes, coaches, teachers, and parents, while ensuring variation in sport type, gender, geography, and school level. This strategy supported both informational depth and case diversity, consistent with the study’s goal of understanding the structural complexity of dual career environments.

To strengthen reliability, triangulation strategies were used. Interview data were compared with national and international policy documents and prior literature on dual careers. Reflexive memo writing during the analysis helped monitor interpretive bias. In addition, three experts in sport education and sociology conducted peer debriefing to review the coding structure, thematic decisions, and analytic interpretations. These steps enhanced the study’s interpretive credibility and theoretical coherence.

**Figure 1:**
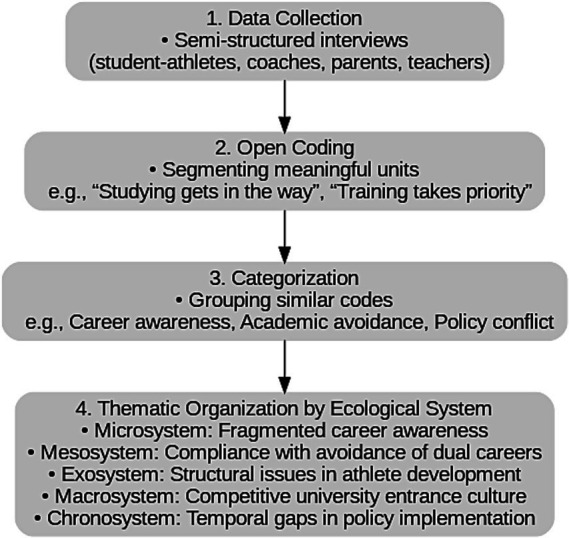
Summary of qualitative analysis procedures based on the ecological systems framework. The figure presents the sequential steps of coding and theme development from data collection to thematic classification across five ecological levels.

Based on the above coding and recategorization process, this study identified five core themes, each corresponding to a level within the ecological systems framework. The overall analytical process is summarized in Figure 1.

## 4 Results

This study applied Bronfenbrenner’s (1979) ecological systems theory as an analytical framework to explore the psychosocial and structural barriers experienced by Korean secondary school student-athletes in dual-career environments. The interview data were analyzed using open and axial coding, and five themes were derived based on the five ecological levels. Each theme was classified using specific analytical criteria based on empirical evidence.

First, the microsystem-level theme—fragmented career awareness—captured student-athletes’ internalized career perceptions and attitudes toward academics. These perceptions were not solely self-formed; rather, they were shaped through daily interactions with coaches and parents, reinforcing a narrow, performance-driven outlook.

Second, the mesosystem-level theme—compliance with the avoidance of dual careers—emerged from the interaction patterns among schools, families, and sports teams. These patterns reflected the failure to coordinate stakeholder demands, resulting in weakened structural support for academic engagement. Teachers, coaches, and parents often conform to a sports-centered environment, lowering institutional expectations for dual-career development.

Third, the exosystem-level theme—structural limitations in the athlete development system—focused on indirect constraints such as competition schedules, university admissions systems, and policy operations. These external factors reinforced performance-first structures and evaluation systems, limiting opportunities for academic participation.

Fourth, the macrosystem-level theme—intensified academic competition in the Korean society—highlighted how South Korea’s hypercompetitive entrance exam culture shapes student-athletes’ experiences. The social expectations of academic achievement, even among athletes, impose dual pressures on them to excel both academically and athletically. This reflects the broader structural tendency to conflate sports success with educational prestige and career stability.

Finally, the chronosystem-level theme—temporal gaps in dual-career policy implementation—identified how mismatches between policy rollout and field-level adaptation create confusion and disadvantages for student-athletes. Disparities in policy timing, generational transitions, and institutional readiness hinder effective adjustments to the dual-career reforms.

By maintaining the theoretical structure while grounding each theme in the participants’ narratives and contextual analysis, this study established distinct criteria for classifying each ecological level. This approach minimized conceptual overlap and ensured empirical clarity in distinguishing each layer of influence.

### 4.1 Microsystem: fragmented career awareness

The theme of fragmented career awareness appeared most prominently at the microsystem level. Ten participants—five student-athletes, three coaches, and two parents—made statements aligned with this theme. They commonly viewed becoming a professional athlete as either the sole or primary career goal. This belief often led them to marginalize academic engagement and school life, treating them as secondary or even as obstacles to athletic success.

Student-athletes perceived training as more beneficial to their future than academic activities and generally showed low motivation for schoolwork. For example, Student-Athlete 1 stated, “Practicing one more serve in the gym helps me more than studying,” and added, “Even if I fully focus on sports, making it pro is not guaranteed—doing both feels like giving up on becoming an athlete.” These statements reflect a negative perception of dual careers, where academic and athletic commitments are seen as incompatible.

Notably, this perception was not solely the result of individual reasoning. It was strongly influenced by coaches and parents. Coach 2 explained, “Student-athletes are preparing to become professionals, so applying the same academic expectations as regular students is inappropriate.” Similarly, Parent 1 stated, “Focusing on sports is more beneficial for my child’s future than studying.” These views reinforced a career identity centered on athletic success and devalued academic development.

Overall, these microsystem-level interactions reduced the support and flexibility student-athletes needed to engage meaningfully in education. As a result, they fostered negative attitudes toward dual careers and reinforced the structural devaluation of academic participation.

### 4.2 Mesosystem: compliance with the avoidance of dual careers

The theme of compliance with the avoidance of dual careers emerged prominently at the mesosystem level. Nine participants—three teachers, three coaches, and three parents—shared experiences that reflected this pattern. The mesosystem refers to the interface between microsystems such as schools, sports teams, and families. It forms a key context where institutional attitudes and structural arrangements toward dual careers are shaped.

The analysis showed that schools often prioritized training schedules over academic engagement to accommodate the demands of coaches and parents. Although teachers recognized the value of classroom participation, they frequently adopted a passive stance under external pressure, favoring accommodation over educational judgment. For example, Teacher 4 stated, “I have no choice but to excuse students from class because of strong parental demands,” and Teacher 3 similarly remarked, “It’s not educationally ideal, but I have to go along with what they ask.”

This tendency also appeared in coach–parent interactions. Parents, driven by performance outcomes and university admissions goals, pushed for extended training time. In response, coaches prioritized athletic training over class attendance. Coach 1 noted, “To improve the athletes’ skills, I have no choice but to extend training hours.”

In this environment, teachers, coaches, and parents collectively conformed to a sport-dominant structure rather than supporting dual careers. This institutional compliance contributed to structural barriers that hindered student-athletes’ efforts to balance school and sport.

### 4.3 Exosystem: structural limitations in the athlete development system

At the exosystem level, participants identified recurring structural issues that restricted student-athletes’ participation in academic activities. Ten participants—three student-athletes, three teachers, two coaches, and two parents—emphasized that Korea’s performance-oriented system, where athletic success strongly influences school admission and career continuity, undermines dual career efforts.

Participants described how the elite sports environment pressured student-athletes to prioritize performance over academics, which reduced their motivation for school. Student-Athlete 5 stated, “I’ve spent so much time just doing sports that if I quit now, I would not be able to do anything else.” Teacher 5 similarly noted, “Many parents demand more training if the student has not achieved podium finishes, since otherwise college admission becomes difficult.” These responses suggest that performance-based advancement leads both athletes and stakeholders to favor training and competition over education.

Several participants also pointed to external systems and schedules as barriers to academic engagement. For instance, Student-Athlete 1 said, “When competitions take place during the school term, I have no choice but to miss class.” Student-Athlete 3 added, “Since there’s an official system allowing me to skip classes, I naturally choose to train.” The conflict between sports schedules and school calendars—combined with policies that normalize absences—deepened the structural disadvantage for student-athletes seeking academic continuity.

This issue also relates to early specialization. Most student-athletes began intensive training in early elementary school, entering environments that prioritized sports over academics from a young age. As a result, they struggled to form consistent study habits or engage meaningfully in school life. Although the Korean government introduced policies such as the “Minimum Academic Achievement System” and the “Advanced School Sports Team Management Framework,” participants widely viewed these initiatives as ineffective in practice.

Taken together, these findings show that Korea’s elite sports system creates structural conditions that threaten the viability of dual careers. At the exosystem level, institutional policies and schedules significantly limit student-athletes’ access to academic opportunities.

### 4.4 Macrosystem: intensified academic competition in Korean society

At the macrosystem level, Korea’s education-centered culture and highly competitive university entrance system strongly influenced student-athletes’ career decisions and dual career paths. Seven participants—two student-athletes, three parents, and two teachers—addressed this issue. They consistently noted that the pressure to gain admission to top universities also applied to student-athletes and caused significant stress during their dual career process.

Student-athletes emphasized that, despite their commitment to sports, they could not neglect the goal of entering a prestigious university. Student-Athlete 1 stated, “We also need to attend good schools, just like regular students,” suggesting that a reputable academic background was essential—even if they left sport. Parent 5 similarly remarked, “If a sports career does not work out, at least they should graduate from a good school,” reinforcing the belief that academic credentials are critical for future job stability.

These statements reflect how, in Korean society, education—especially university admission—serves as a major path to social mobility. This expectation also applies to student-athletes, who must succeed in both athletic and academic competition. As a result, they often experience increased physical and mental stress from dual demands.

In addition, elite university admission is seen as a pathway to joining strong semi-professional or professional teams, or ensuring career stability after sports. This dual pressure—to maintain academic performance while training intensively—often left student-athletes and their parents struggling to balance both goals.

These findings indicate that Korea’s intense academic competition is deeply embedded in the sports domain. It functions as a structural force that shapes dual career environments and amplifies systemic pressure in both education and athletics.

### 4.5 Chronosystem: temporal gaps in dual career policy implementation

The chronosystem refers to the temporal dimension of development and examines how policy and system changes interact with individuals over time. In this study, participants raised concerns about the misalignment between the introduction of dual career policies and their on-site implementation. Five participants—two parents, two coaches, and one teacher—recognized the value of these policies but noted that delays in field-level adaptation could structurally disadvantage student-athletes.

Participants specifically worried that athletes who had planned their careers under the previous sport-centered system would struggle to meet new academic requirements—such as minimum academic standards and attendance rules—introduced by the reforms. Parent 1 warned, “If we suddenly expect kids who have only trained to now fully attend classes, the burden falls entirely on the student.” Coach 4 similarly cautioned, “At this rate, they will not be able to compete or get into university,” questioning the pace and manner of enforcement.

While most participants agreed on the legitimacy and need for dual career reform, they stressed the importance of long-term transition planning and staggered implementation by age group. Poor field-level preparation, weak communication of institutional changes, and individual differences in student-athlete capacity all contributed to the difficulty of adapting.

These findings show that not only the content but also the timing and method of policy implementation significantly affect student-athletes’ ability to adjust to dual career systems. Middle and high school athletes, in particular, were seen as the most vulnerable to negative consequences from abrupt institutional shifts.

**Figure 2:**
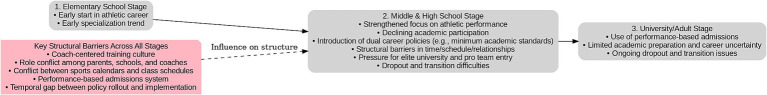
Conceptual diagram of dual career pathways and structural barriers among Korean student-athletes. This figure illustrates key transition points from elementary school to adulthood and highlights recurring structural constraints affecting dual career development.

Building on the above analysis, Figure 2 presents an integrated overview of the dual-career trajectories of Korean student-athletes and the structural barriers shaping them. The diagram illustrates the key transition points from elementary school to university and adulthood, while also mapping the structural constraints operating in parallel across each developmental stage. This visual framework captures the complex and layered context of dual careers.

## 5 Discussion

This study applied Bronfenbrenner’s ecological systems theory to conduct a multilayered analysis of the dual career environment of Korean student-athletes. The main findings at each ecological level are summarized below.

First, at the microsystem level, student-athletes held narrowly defined career perceptions, strongly shaped by coaches and parents. These influences led them to view academics as secondary and becoming a professional athlete as their only goal. This aligns with findings from Stephan and Brewer (2007) and Sum et al. (2017), who emphasized the impact of significant others on career decisions. Cabrita et al. (2014) similarly noted that emotional support from parents and coaches plays a critical role in adolescent athletes’ career development—consistent with this study’s results.

Second, in the mesosystem, interactions among teachers, coaches, and parents reflected a conformist attitude toward avoiding dual careers. Although teachers are responsible for protecting students’ right to learn, they often failed to play an active role due to conflicting stakeholder demands. This supports Tekavc et al.’s (2015) concept of “role conflict.” De Brandt et al. (2017) also reported that poor stakeholder collaboration impedes student-athletes’ ability to sustain dual careers—a finding echoed in this study. Kiens and Larsen (2021) further described how Estonian high school athletes, lacking institutional support, must independently manage both academics and training. This highlights the structural limitations of Korea’s individually driven mesosystem responses.

Third, at the exosystem level, structural weaknesses in Korea’s student-athlete development system became evident. The admission system relies heavily on athletic performance, creating external constraints on academic engagement. This limits career diversification and reflects a misalignment between entrance policies and dual career support. Aquilina (2013) stressed the importance of academic involvement for holistic development and post-retirement readiness, warning against sport-centered systems. This study reinforces that view, showing how Korea’s performance-based admissions restrict academic and long-term career opportunities. In contrast, Linnér et al. (2020) described Sweden’s “request-based support system,” which respects athlete autonomy and improves both flexibility and institutional accountability—starkly different from Korea’s rigid, directive model.

Fourth, at the macrosystem level, Korea’s intense exam culture makes it even harder for student-athletes to balance sports and academics. Parental expectations and social norms often push them toward a singular path: university admission through athletic achievement. These findings support Guidotti et al.’s (2015) typology, which emphasizes cultural background as a key factor in dual career acceptance. Stambulova and Wylleman (2019) also stressed the importance of culturally adapted support systems—closely aligned with this study’s observations.

Fifth, the chronosystem analysis revealed that sudden policy changes created adaptation problems for student-athletes accustomed to the previous training-centered system. These transitions caused confusion and disadvantage, highlighting the need for gradual policy implementation. De Maio et al. (2025) similarly noted that time lags between policy design and execution hinder acceptance of dual careers—an effect confirmed by this study. These timing discrepancies are not merely issues of policy implementation speed or direction; rather, they reflect a complex outcome stemming from the lack of field-level adaptability and structural responsiveness.

The rapid expansion of digital learning environments and hybrid education structures in the post-pandemic era has emerged as not a temporary solution but a practical means of supporting dual-career pathways for student-athletes. As confirmed in this study, the current system tends to place the burden of adjustment on individual student-athletes in the face of rapid institutional changes, highlighting the need for structural flexibility and contextual support. Therefore, digital and flexible learning formats should be integrated into a sustainable, non-disruptive framework for dual-career support. This is especially relevant in the Korean context, where the training-centered school system and university entrance-oriented culture coexist. In such settings, these formats may serve as practical alternatives to compensate for the systemic limitations.

To ensure the sustainability of dual-career environments, it is essential to move beyond isolated institutional reforms and establish a comprehensive response system through coordination and integration across all ecological levels. Together, these findings show that improving dual career environments requires coordination across all ecological levels. This includes expanding career options in the microsystem, fostering collaboration in the mesosystem, increasing institutional flexibility in the exosystem, enhancing cultural acceptance in the macrosystem, and phasing in reforms over time within the chronosystem. Only an integrated approach can create a sustainable dual career system for student-athletes in Korea.

## 6 Conclusion

This study conducted a multilayered analysis of the psychosocial and structural barriers in the dual career environments of Korean student-athletes, using Bronfenbrenner’s ecological systems theory as its framework. The findings revealed fragmented career awareness at the microsystem level, coordination failures among stakeholders at the mesosystem level, institutional misalignment at the exosystem level, sociocultural pressure at the macrosystem level, and timing gaps in policy implementation at the chronosystem level. These results suggest that student-athletes’ challenges in balancing academics and athletics stem from complex interactions across ecological layers.

Improving the dual career environment requires not isolated reforms, but comprehensive, multi-level strategies tailored to each ecological system. Key areas for action include promoting flexible career identity development, building systems for institutional coordination, shifting cultural perceptions of dual careers, and implementing policy transitions gradually over time.

### 6.1 Policy recommendations and practical implications

Based on the findings and discussion, the following policy recommendations and practical implications are proposed to improve the dual career environment for Korean student-athletes:

First, a multidimensional approach is needed to broaden student-athletes’ career perceptions. Currently, career aspirations focus too narrowly on becoming professional athletes, which reduces motivation for academics. To address this, schools should offer broader educational initiatives, expand career counseling, and provide exposure to diverse professions that help build multifaceted identities.

Second, the current admissions system, centered on athletic merit, should be restructured to institutionalize a dual-track model that values both academic and athletic achievement. University entrance criteria should include academic performance and student records, not just athletic results. Clear academic policies must guarantee student-athletes’ right to education, thereby increasing their motivation and participation.

Third, a collaborative governance model should be established among teachers, coaches, and parents to support dual careers in daily settings. This includes creating communication councils, coordinating school and training schedules, and assigning personnel dedicated to dual career support. These structures can reduce stakeholder conflict and foster balanced development.

Fourth, phased implementation is essential when introducing new dual career policies. Minimum academic requirements, for example, should be introduced gradually, especially for students trained under the previous system. Pilot programs, age-based timelines, and continuous evaluation and revision are needed to ensure smooth transitions.

Fifth, international best practices—such as the EU Dual Career Guidelines, the NCAA model in the U. S., and Sweden’s RIG (National Sports Schools)—should be localized to fit Korea’s cultural and institutional context. Adaptations may include flexible scheduling, mobile learning platforms, and hybrid instruction. Localized approaches must consider cultural acceptance and system feasibility.

In sum, improving dual career conditions for student-athletes requires comprehensive restructuring—not incremental adjustments. Sustainable sports policy must enable student-athletes to pursue diverse life paths, rather than locking them into a single, performance-driven trajectory.

### 6.2 Limitations and future directions

This study provides a meaningful qualitative exploration of dual career experiences among Korean secondary school student-athletes. However, several limitations should be noted.

First, the sample focused on specific sports disciplines, which may limit the representation of regional and sport-specific differences. Second, as with most qualitative research, the findings are not generalizable, and the interpretive analysis may include inherent subjectivity. Third, the use of one-time, in-depth interviews limited the ability to capture long-term changes in student-athletes’ dual career paths.

Future research should include a wider range of regions, sports disciplines, school levels (elementary, middle, and high school), and gender comparisons to enhance contextual understanding. Incorporating quantitative data through mixed-methods or longitudinal designs could help validate the policy recommendations proposed in this study. Follow-up studies that assess the impact and acceptance of implemented policies would also provide stronger evidence for institutional reform. Finally, international comparative research could offer valuable cross-cultural insights into Korea’s unique dual career structures and limitations.

### 6.3 Ethics statement

This study did not involve surveys, biometric data, or diagnostic information, and the interview process posed minimal physical or psychological risk to participants. All participants voluntarily consented after receiving a full explanation of the study’s purpose, procedures, audio recording, and data anonymization. Written informed consent was obtained from each participant. For minor student-athletes, additional consent was obtained from their legal guardians. Participants were also informed that they could withdraw from the interview at any time without penalty.

No personally identifiable information—such as names, affiliations, or contact details—was collected. All data were anonymized and stored in encrypted files, and will be securely deleted after being retained for a designated period following the study’s conclusion. This research was conducted in accordance with the Declaration of Helsinki and Article 33 of the Enforcement Decree of the Bioethics and Safety Act of the Republic of Korea, which defines exemption criteria for ethics review. Therefore, this study qualifies for exemption from formal ethics committee approval.

Generative AI tools (e.g., ChatGPT by OpenAI) were used in a limited capacity to refine the language and phrasing of selected sentences during manuscript preparation. The author is fully responsible for conducting the study’s design, data analysis, interpretation, and theoretical development. All findings and conclusions reflect the author’s original work and judgment. No artificial intelligence tools were used to generate factual content or perform any aspect of the research analysis.

## Data Availability

The original contributions presented in the study are included in the article/supplementary material, further inquiries can be directed to the corresponding author.
